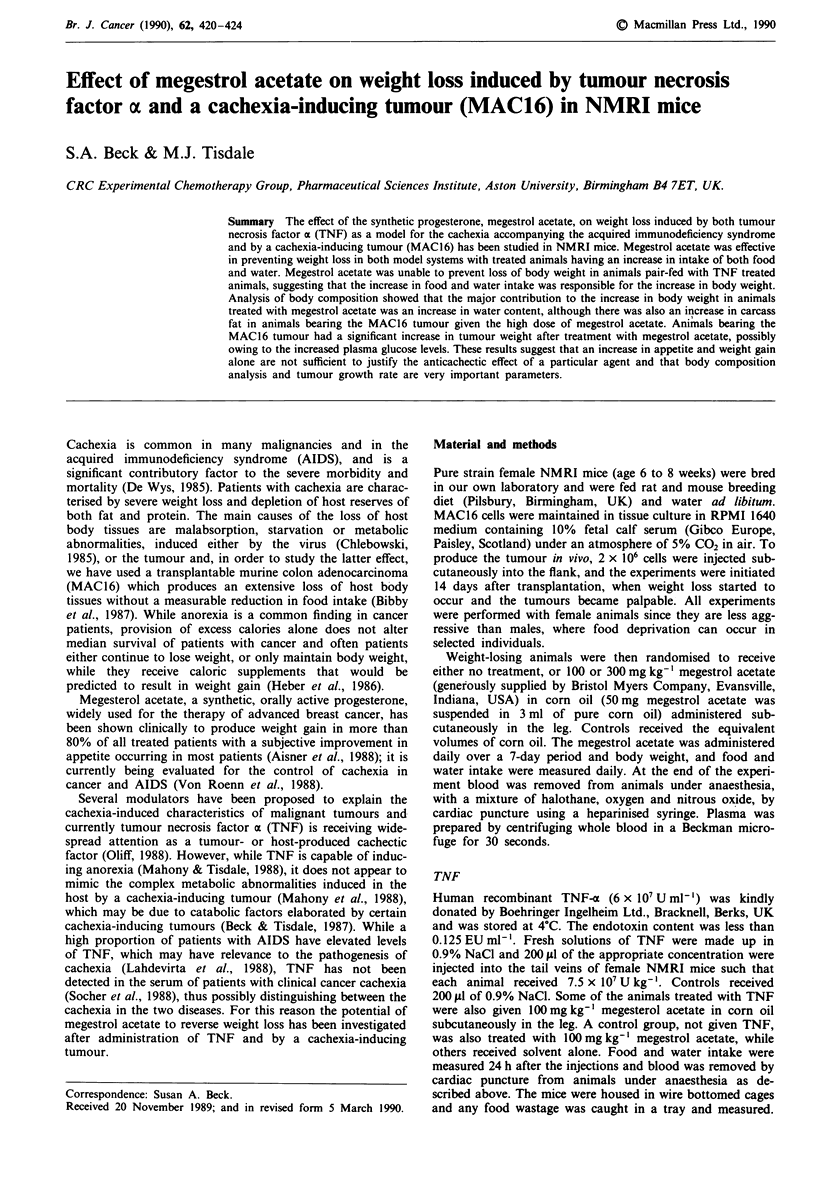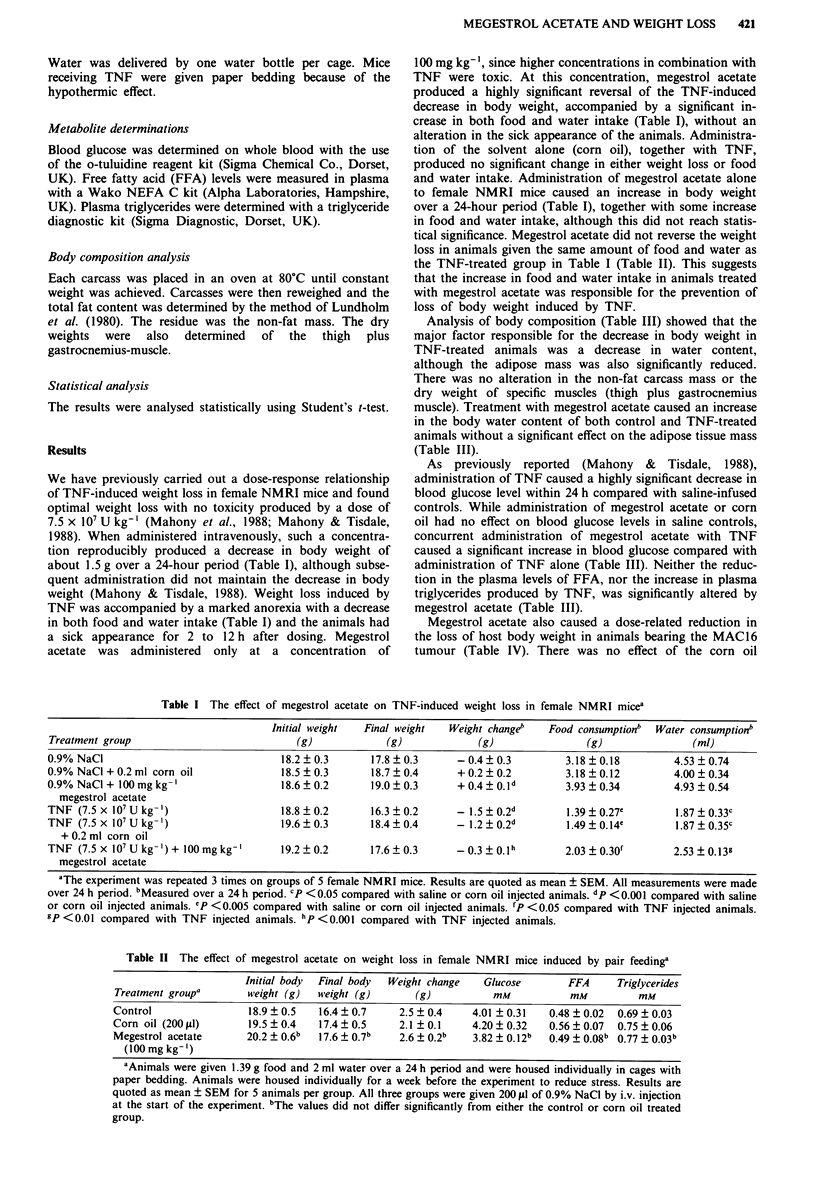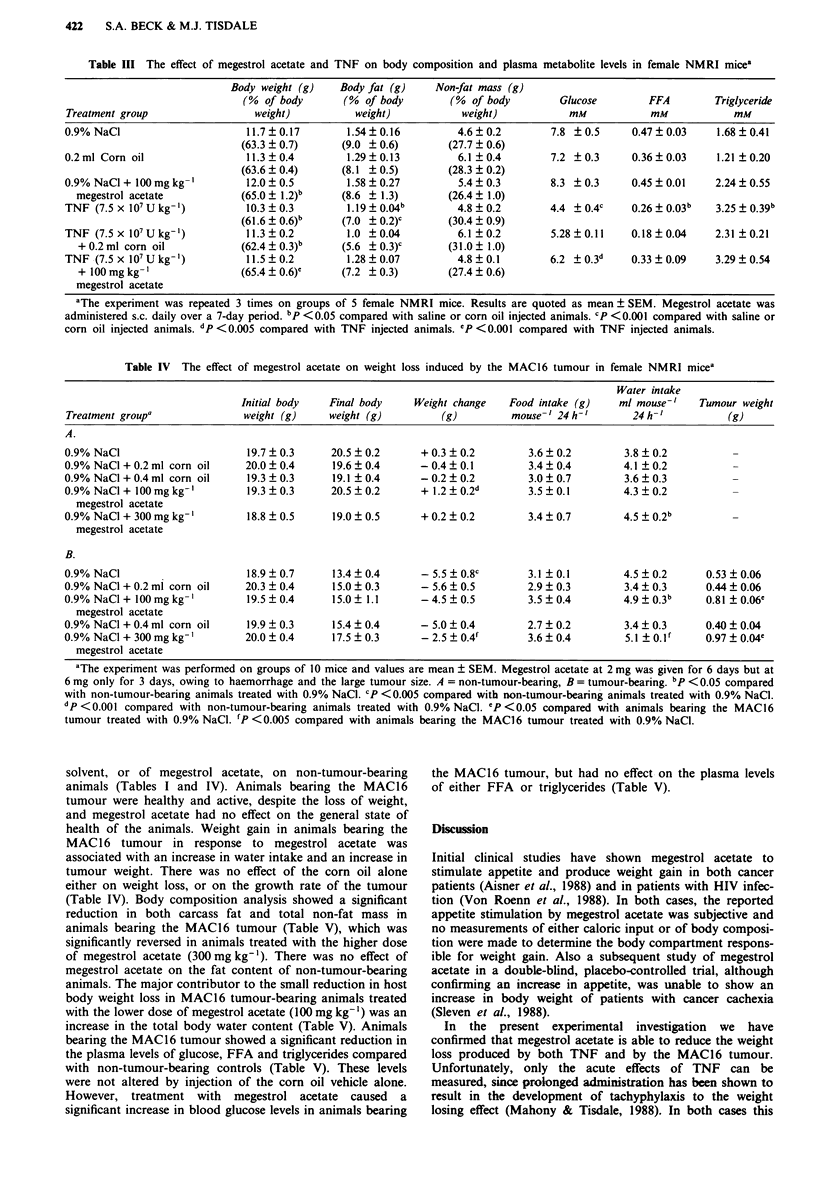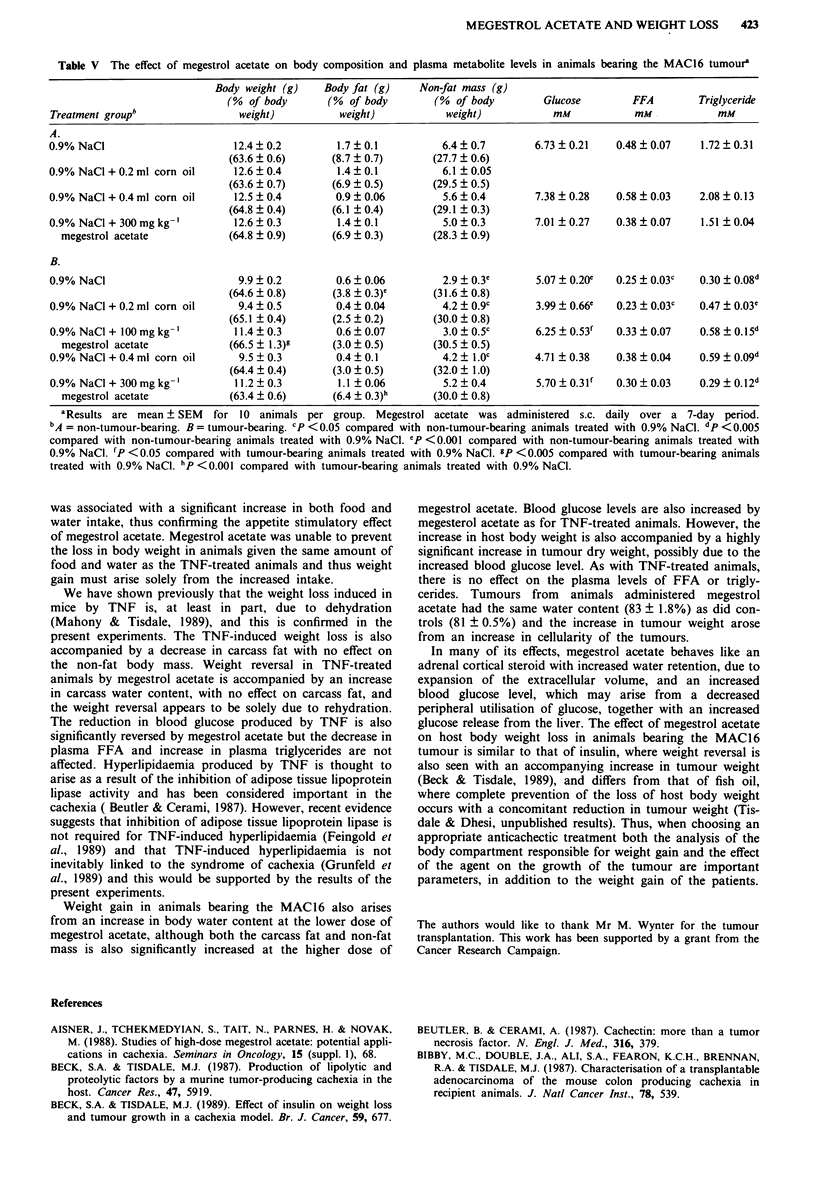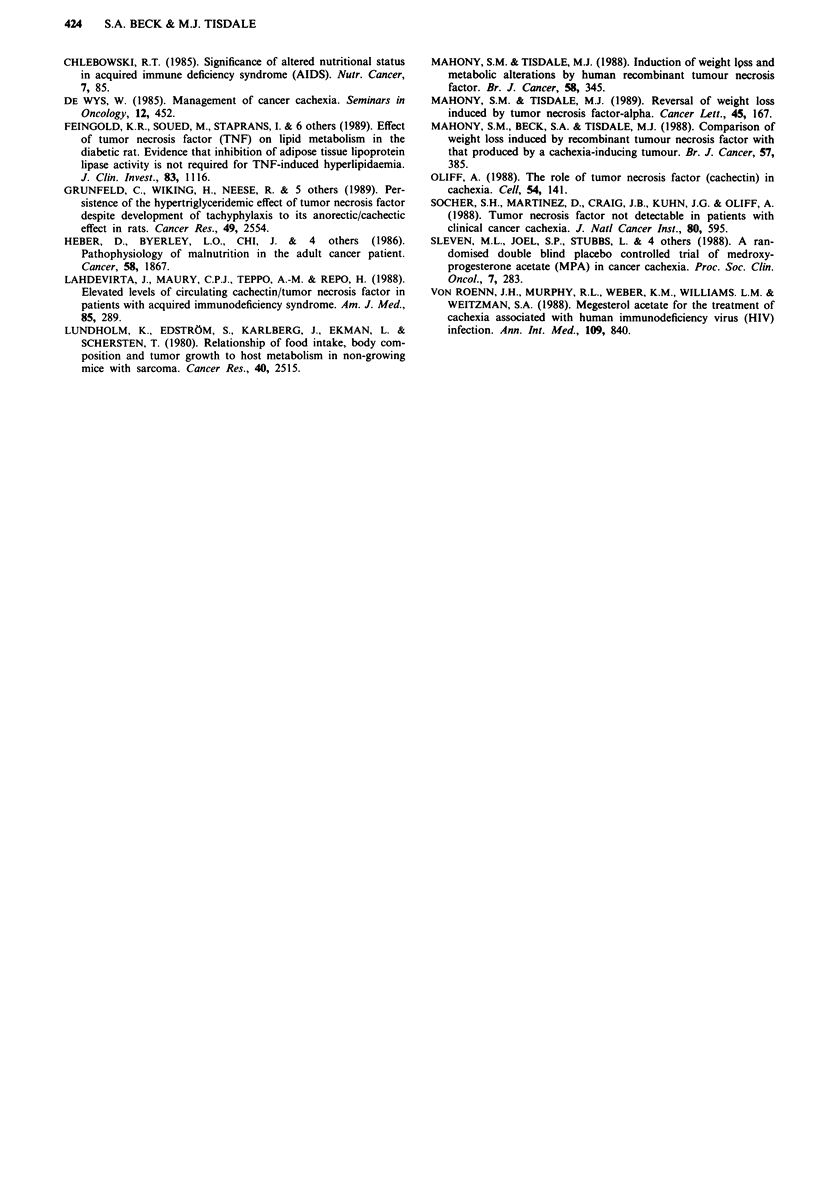# Effect of megestrol acetate on weight loss induced by tumour necrosis factor alpha and a cachexia-inducing tumour (MAC16) in NMRI mice.

**DOI:** 10.1038/bjc.1990.310

**Published:** 1990-09

**Authors:** S. A. Beck, M. J. Tisdale

**Affiliations:** CRC Experimental Chemotherapy Group, Aston University, Birmingham, UK.

## Abstract

The effect of the synthetic progesterone, megestrol acetate, on weight loss induced by both tumour necrosis factor alpha (TNF) as a model for the cachexia accompanying the acquired immunodeficiency syndrome and by a cachexia-inducing tumour (MAC16) has been studied in NMRI mice. Megestrol acetate was effective in preventing weight loss in both model systems with treated animals having an increase in intake of both food and water. Megestrol acetate was unable to prevent loss of body weight in animals pair-fed with TNF treated animals, suggesting that the increase in food and water intake was responsible for the increase in body weight. Analysis of body composition showed that the major contribution to the increase in body weight in animals treated with megestrol acetate was an increase in water content, although there was also an increase in carcass fat in animals bearing the MAC16 tumour given the high dose of megestrol acetate. Animals bearing the MAC16 tumour had a significant increase in tumour weight after treatment with megestrol acetate, possibly owing to the increased plasma glucose levels. These results suggest that an increase in appetite and weight gain alone are not sufficient to justify the anticachectic effect of a particular agent and that body composition analysis and tumour growth rate are very important parameters.


Br. J. Cancer (1990), 62, 420-424 © Macmillan Press Ltd., 1990

### Effect of megestrol acetate on weight loss induced by tumour necrosis factor α and a cachexia-inducing tumour (MAC16) in NMRI mice

S.A. Beck & M.J. Tisdale

*CRC Experimental Chemotherapy Group, Pharmaceutical Sciences Institute, Aston University, Birmingham B4 7ET, UK.*

**Summary** The effect of the synthetic progesterone, megestrol acetate, on weight loss induced by both tumour necrosis factor α (TNF) as a model for the cachexia accompanying the acquired immunodeficiency syndrome and by a cachexia-inducing tumour (MAC16) has been studied in NMRI mice. Megestrol acetate was effective in preventing weight loss in both model systems with treated animals having an increase in intake of both food and water. Megestrol acetate was unable to prevent loss of body weight in animals pair-fed with TNF treated animals, suggesting that the increase in food and water intake was responsible for the increase in body weight. Analysis of body composition showed that the major contribution to the increase in body weight in animals treated with megestrol acetate was an increase in water content, although there was also an increase in carcass fat in animals bearing the MAC16 tumour given the high dose of megestrol acetate. Animals bearing the MAC16 tumour had a significant increase in tumour weight after treatment with megestrol acetate, possibly owing to the increased plasma glucose levels. These results suggest that an increase in appetite and weight gain alone are not sufficient to justify the anticachectic effect of a particular agent and that body composition analysis and tumour growth rate are very important parameters.

Cachexia is common in many malignancies and in the acquired immunodeficiency syndrome (AIDS), and is a significant contributory factor to the severe morbidity and mortality (De Wys, 1985). Patients with cachexia are characterised by severe weight loss and depletion of host reserves of both fat and protein. The main causes of the loss of host body tissues are malabsorption, starvation or metabolic abnormalities, induced either by the virus (Chlebowski, 1985), or the tumour and, in order to study the latter effect, we have used a transplantable murine colon adenocarcinoma (MAC16) which produces an extensive loss of host body tissues without a measurable reduction in food intake (Bibby *et al.*, 1987). While anorexia is a common finding in cancer patients, provision of excess calories alone does not alter median survival of patients with cancer and often patients either continue to lose weight, or only maintain body weight, while they receive caloric supplements that would be predicted to result in weight gain (Heber *et al.*, 1986).

Megesterol acetate, a synthetic, orally active progesterone, widely used for the therapy of advanced breast cancer, has been shown clinically to produce weight gain in more than 80% of all treated patients with a subjective improvement in appetite occurring in most patients (Aisner *et al.*, 1988); it is currently being evaluated for the control of cachexia in cancer and AIDS (Von Roenn *et al.*, 1988).

Several modulators have been proposed to explain the cachexia-induced characteristics of malignant tumours and currently tumour necrosis factor α (TNF) is receiving widespread attention as a tumour- or host-produced cachectic factor (Oliff, 1988). However, while TNF is capable of inducing anorexia (Mahony & Tisdale, 1988), it does not appear to mimic the complex metabolic abnormalities induced in the host by a cachexia-inducing tumour (Mahony *et al.*, 1988), which may be due to catabolic factors elaborated by certain cachexia-inducing tumours (Beck & Tisdale, 1987). While a high proportion of patients with AIDS have elevated levels of TNF, which may have relevance to the pathogenesis of cachexia (Lahdevirta *et al.*, 1988), TNF has not been detected in the serum of patients with clinical cancer cachexia (Socher *et al.*, 1988), thus possibly distinguishing between the cachexia in the two diseases. For this reason the potential of megestrol acetate to reverse weight loss has been investigated after administration of TNF and by a cachexia-inducing tumour.

Correspondence: Susan A. Beck.
Received 20 November 1989; and in revised form 5 March 1990.

### Material and methods

Pure strain female NMRI mice (age 6 to 8 weeks) were bred in our own laboratory and were fed rat and mouse breeding diet (Pilsbury, Birmingham, UK) and water *ad libitum*. MAC16 cells were maintained in tissue culture in RPMI 1640 medium containing 10% fetal calf serum (Gibco Europe, Paisley, Scotland) under an atmosphere of 5% $CO_2$ in air. To produce the tumour *in vivo*, $2 \times 10^6$ cells were injected subcutaneously into the flank, and the experiments were initiated 14 days after transplantation, when weight loss started to occur and the tumours became palpable. All experiments were performed with female animals since they are less aggressive than males, where food deprivation can occur in selected individuals.

Weight-losing animals were then randomised to receive either no treatment, or 100 or 300 mg $kg^{-1}$ megestrol acetate (generously supplied by Bristol Myers Company, Evansville, Indiana, USA) in corn oil (50 mg megestrol acetate was suspended in 3 ml of pure corn oil) administered subcutaneously in the leg. Controls received the equivalent volumes of corn oil. The megestrol acetate was administered daily over a 7-day period and body weight, and food and water intake were measured daily. At the end of the experiment blood was removed from animals under anaesthesia, with a mixture of halothane, oxygen and nitrous oxide, by cardiac puncture using a heparinised syringe. Plasma was prepared by centrifuging whole blood in a Beckman microfuge for 30 seconds.

### *TNF*

Human recombinant TNF-α ($6 \times 10^7$ U $ml^{-1}$) was kindly donated by Boehringer Ingelheim Ltd., Bracknell, Berks, UK and was stored at 4°C. The endotoxin content was less than 0.125 EU $ml^{-1}$. Fresh solutions of TNF were made up in 0.9% NaCl and 200 μl of the appropriate concentration were injected into the tail veins of female NMRI mice such that each animal received $7.5 \times 10^7$ U $kg^{-1}$. Controls received 200 μl of 0.9% NaCl. Some of the animals treated with TNF were also given 100 mg $kg^{-1}$ megesterol acetate in corn oil subcutaneously in the leg. A control group, not given TNF, was also treated with 100 mg $kg^{-1}$ megestrol acetate, while others received solvent alone. Food and water intake were measured 24 h after the injections and blood was removed by cardiac puncture from animals under anaesthesia as described above. The mice were housed in wire bottomed cages and any food wastage was caught in a tray and measured.

Water was delivered by one water bottle per cage. Mice receiving TNF were given paper bedding because of the hypothermic effect.

*Metabolite determinations*

Blood glucose was determined on whole blood with the use of the o-tuluidine reagent kit (Sigma Chemical Co., Dorset, UK). Free fatty acid (FFA) levels were measured in plasma with a Wako NEFA C kit (Alpha Laboratories, Hampshire, UK). Plasma triglycerides were determined with a triglyceride diagnostic kit (Sigma Diagnostic, Dorset, UK).

*Body composition analysis*

Each carcass was placed in an oven at 80°C until constant weight was achieved. Carcasses were then reweighed and the total fat content was determined by the method of Lundholm *et al.* (1980). The residue was the non-fat mass. The dry weights were also determined of the thigh plus gastrocnemius-muscle.

*Statistical analysis*

The results were analysed statistically using Student's *t*-test.

**Results**

We have previously carried out a dose-response relationship of TNF-induced weight loss in female NMRI mice and found optimal weight loss with no toxicity produced by a dose of $7.5 \times 10^7$ U $kg^{-1}$ (Mahony *et al.*, 1988; Mahony & Tisdale, 1988). When administered intravenously, such a concentration reproducibly produced a decrease in body weight of about 1.5 g over a 24-hour period (Table I), although subsequent administration did not maintain the decrease in body weight (Mahony & Tisdale, 1988). Weight loss induced by TNF was accompanied by a marked anorexia with a decrease in both food and water intake (Table I) and the animals had a sick appearance for 2 to 12 h after dosing. Megestrol acetate was administered only at a concentration of 100 mg $kg^{-1}$, since higher concentrations in combination with TNF were toxic. At this concentration, megestrol acetate produced a highly significant reversal of the TNF-induced decrease in body weight, accompanied by a significant increase in both food and water intake (Table I), without an alteration in the sick appearance of the animals. Administration of the solvent alone (corn oil), together with TNF, produced no significant change in either weight loss or food and water intake. Administration of megestrol acetate alone to female NMRI mice caused an increase in body weight over a 24-hour period (Table I), together with some increase in food and water intake, although this did not reach statistical significance. Megestrol acetate did not reverse the weight loss in animals given the same amount of food and water as the TNF-treated group in Table I (Table II). This suggests that the increase in food and water intake in animals treated with megestrol acetate was responsible for the prevention of loss of body weight induced by TNF.

Analysis of body composition (Table III) showed that the major factor responsible for the decrease in body weight in TNF-treated animals was a decrease in water content, although the adipose mass was also significantly reduced. There was no alteration in the non-fat carcass mass or the dry weight of specific muscles (thigh plus gastrocnemius muscle). Treatment with megestrol acetate caused an increase in the body water content of both control and TNF-treated animals without a significant effect on the adipose tissue mass (Table III).

As previously reported (Mahony & Tisdale, 1988), administration of TNF caused a highly significant decrease in blood glucose level within 24 h compared with saline-infused controls. While administration of megestrol acetate or corn oil had no effect on blood glucose levels in saline controls, concurrent administration of megestrol acetate with TNF caused a significant increase in blood glucose compared with administration of TNF alone (Table III). Neither the reduction in the plasma levels of FFA, nor the increase in plasma triglycerides produced by TNF, was significantly altered by megestrol acetate (Table III).

Megestrol acetate also caused a dose-related reduction in the loss of host body weight in animals bearing the MAC16 tumour (Table IV). There was no effect of the corn oil

**Table I** The effect of megestrol acetate on TNF-induced weight loss in female NMRI mice[a]

| Treatment group | Initial weight (g) | Final weight (g) | Weight change[b] (g) | Food consumption[b] (g) | Water consumption[b] (ml) |
|---|---|---|---|---|---|
| 0.9% NaCl | 18.2 ± 0.3 | 17.8 ± 0.3 | − 0.4 ± 0.3 | 3.18 ± 0.18 | 4.53 ± 0.74 |
| 0.9% NaCl + 0.2 ml corn oil | 18.5 ± 0.3 | 18.7 ± 0.4 | + 0.2 ± 0.2 | 3.18 ± 0.12 | 4.00 ± 0.34 |
| 0.9% NaCl + 100 mg $kg^{-1}$ megestrol acetate | 18.6 ± 0.2 | 19.0 ± 0.3 | + 0.4 ± 0.1[d] | 3.93 ± 0.34 | 4.93 ± 0.54 |
| TNF ($7.5 \times 10^7$ U $kg^{-1}$) | 18.8 ± 0.2 | 16.3 ± 0.2 | − 1.5 ± 0.2[d] | 1.39 ± 0.27[e] | 1.87 ± 0.33[c] |
| TNF ($7.5 \times 10^7$ U $kg^{-1}$) + 0.2 ml corn oil | 19.6 ± 0.3 | 18.4 ± 0.4 | − 1.2 ± 0.2[d] | 1.49 ± 0.14[e] | 1.87 ± 0.35[c] |
| TNF ($7.5 \times 10^7$ U $kg^{-1}$) + 100 mg $kg^{-1}$ megestrol acetate | 19.2 ± 0.2 | 17.6 ± 0.3 | − 0.3 ± 0.1[h] | 2.03 ± 0.30[f] | 2.53 ± 0.13[g] |

[a]The experiment was repeated 3 times on groups of 5 female NMRI mice. Results are quoted as mean ± SEM. All measurements were made over 24 h period. [b]Measured over a 24 h period. [c]$P < 0.05$ compared with saline or corn oil injected animals. [d]$P < 0.001$ compared with saline or corn oil injected animals. [e]$P < 0.005$ compared with saline or corn oil injected animals. [f]$P < 0.05$ compared with TNF injected animals. [g]$P < 0.01$ compared with TNF injected animals. [h]$P < 0.001$ compared with TNF injected animals.

**Table II** The effect of megestrol acetate on weight loss in female NMRI mice induced by pair feeding[a]

| Treatment group[a] | Initial body weight (g) | Final body weight (g) | Weight change (g) | Glucose mM | FFA mM | Triglycerides mM |
|---|---|---|---|---|---|---|
| Control | 18.9 ± 0.5 | 16.4 ± 0.7 | 2.5 ± 0.4 | 4.01 ± 0.31 | 0.48 ± 0.02 | 0.69 ± 0.03 |
| Corn oil (200 µl) | 19.5 ± 0.4 | 17.4 ± 0.5 | 2.1 ± 0.1 | 4.20 ± 0.32 | 0.56 ± 0.07 | 0.75 ± 0.06 |
| Megestrol acetate (100 mg $kg^{-1}$) | 20.2 ± 0.6[b] | 17.6 ± 0.7[b] | 2.6 ± 0.2[b] | 3.82 ± 0.12[b] | 0.49 ± 0.08[b] | 0.77 ± 0.03[b] |

[a]Animals were given 1.39 g food and 2 ml water over a 24 h period and were housed individually in cages with paper bedding. Animals were housed individually for a week before the experiment to reduce stress. Results are quoted as mean ± SEM for 5 animals per group. All three groups were given 200 µl of 0.9% NaCl by i.v. injection at the start of the experiment. [b]The values did not differ significantly from either the control or corn oil treated group.

**Table III** The effect of megestrol acetate and TNF on body composition and plasma metabolite levels in female NMRI mice[a]

| Treatment group | *Body weight (g) (% of body weight)* | *Body fat (g) (% of body weight)* | *Non-fat mass (g) (% of body weight)* | *Glucose mM* | *FFA mM* | *Triglyceride mM* |
|---|---|---|---|---|---|---|
| 0.9% NaCl | 11.7 ± 0.17 (63.3 ± 0.7) | 1.54 ± 0.16 (9.0 ± 0.6) | 4.6 ± 0.2 (27.7 ± 0.6) | 7.8 ± 0.5 | 0.47 ± 0.03 | 1.68 ± 0.41 |
| 0.2 ml Corn oil | 11.3 ± 0.4 (63.6 ± 0.4) | 1.29 ± 0.13 (8.1 ± 0.5) | 6.1 ± 0.4 (28.3 ± 0.2) | 7.2 ± 0.3 | 0.36 ± 0.03 | 1.21 ± 0.20 |
| 0.9% NaCl + 100 mg kg$^{-1}$ megestrol acetate | 12.0 ± 0.5 (65.0 ± 1.2)[b] | 1.58 ± 0.27 (8.6 ± 1.3) | 5.4 ± 0.3 (26.4 ± 1.0) | 8.3 ± 0.3 | 0.45 ± 0.01 | 2.24 ± 0.55 |
| TNF (7.5 x 10$^7$ U kg$^{-1}$) | 10.3 ± 0.3 (61.6 ± 0.6)[b] | 1.19 ± 0.04[b] (7.0 ± 0.2)[c] | 4.8 ± 0.2 (30.4 ± 0.9) | 4.4 ± 0.4[c] | 0.26 ± 0.03[b] | 3.25 ± 0.39[b] |
| TNF (7.5 x 10$^7$ U kg$^{-1}$) + 0.2 ml corn oil | 11.3 ± 0.2 (62.4 ± 0.3)[b] | 1.0 ± 0.04 (5.6 ± 0.3)[c] | 6.1 ± 0.2 (31.0 ± 1.0) | 5.28 ± 0.11 | 0.18 ± 0.04 | 2.31 ± 0.21 |
| TNF (7.5 x 10$^7$ U kg$^{-1}$) + 100 mg kg$^{-1}$ megestrol acetate | 11.5 ± 0.2 (65.4 ± 0.6)[e] | 1.28 ± 0.07 (7.2 ± 0.3) | 4.8 ± 0.1 (27.4 ± 0.6) | 6.2 ± 0.3[d] | 0.33 ± 0.09 | 3.29 ± 0.54 |

[a]The experiment was repeated 3 times on groups of 5 female NMRI mice. Results are quoted as mean ± SEM. Megestrol acetate was administered s.c. daily over a 7-day period. [b]$P < 0.05$ compared with saline or corn oil injected animals. [c]$P < 0.001$ compared with saline or corn oil injected animals. [d]$P < 0.005$ compared with TNF injected animals. [e]$P < 0.001$ compared with TNF injected animals.

**Table IV** The effect of megestrol acetate on weight loss induced by the MAC16 tumour in female NMRI mice[a]

| Treatment group[a] | *Initial body weight (g)* | *Final body weight (g)* | *Weight change (g)* | *Food intake (g) mouse$^{-1}$ 24 h$^{-1}$* | *Water intake ml mouse$^{-1}$ 24 h$^{-1}$* | *Tumour weight (g)* |
|---|---|---|---|---|---|---|
| *A.* | | | | | | |
| 0.9% NaCl | 19.7 ± 0.3 | 20.5 ± 0.2 | + 0.3 ± 0.2 | 3.6 ± 0.2 | 3.8 ± 0.2 | – |
| 0.9% NaCl + 0.2 ml corn oil | 20.0 ± 0.4 | 19.6 ± 0.4 | – 0.4 ± 0.1 | 3.4 ± 0.4 | 4.1 ± 0.2 | – |
| 0.9% NaCl + 0.4 ml corn oil | 19.3 ± 0.3 | 19.1 ± 0.4 | – 0.2 ± 0.2 | 3.0 ± 0.7 | 3.6 ± 0.3 | – |
| 0.9% NaCl + 100 mg kg$^{-1}$ megestrol acetate | 19.3 ± 0.3 | 20.5 ± 0.2 | + 1.2 ± 0.2[d] | 3.5 ± 0.1 | 4.3 ± 0.2 | – |
| 0.9% NaCl + 300 mg kg$^{-1}$ megestrol acetate | 18.8 ± 0.5 | 19.0 ± 0.5 | + 0.2 ± 0.2 | 3.4 ± 0.7 | 4.5 ± 0.2[b] | – |
| *B.* | | | | | | |
| 0.9% NaCl | 18.9 ± 0.7 | 13.4 ± 0.4 | – 5.5 ± 0.8[c] | 3.1 ± 0.1 | 4.5 ± 0.2 | 0.53 ± 0.06 |
| 0.9% NaCl + 0.2 ml corn oil | 20.3 ± 0.4 | 15.0 ± 0.3 | – 5.6 ± 0.5 | 2.9 ± 0.3 | 3.4 ± 0.3 | 0.44 ± 0.06 |
| 0.9% NaCl + 100 mg kg$^{-1}$ megestrol acetate | 19.5 ± 0.4 | 15.0 ± 1.1 | – 4.5 ± 0.5 | 3.5 ± 0.4 | 4.9 ± 0.3[b] | 0.81 ± 0.06[e] |
| 0.9% NaCl + 0.4 ml corn oil | 19.9 ± 0.3 | 15.4 ± 0.4 | – 5.0 ± 0.4 | 2.7 ± 0.2 | 3.4 ± 0.3 | 0.40 ± 0.04 |
| 0.9% NaCl + 300 mg kg$^{-1}$ megestrol acetate | 20.0 ± 0.4 | 17.5 ± 0.3 | – 2.5 ± 0.4[f] | 3.6 ± 0.4 | 5.1 ± 0.1[f] | 0.97 ± 0.04[e] |

[a]The experiment was performed on groups of 10 mice and values are mean ± SEM. Megestrol acetate at 2 mg was given for 6 days but at 6 mg only for 3 days, owing to haemorrhage and the large tumour size. *A* = non-tumour-bearing, *B* = tumour-bearing. [b]$P < 0.05$ compared with non-tumour-bearing animals treated with 0.9% NaCl. [c]$P < 0.005$ compared with non-tumour-bearing animals treated with 0.9% NaCl. [d]$P < 0.001$ compared with non-tumour-bearing animals treated with 0.9% NaCl. [e]$P < 0.05$ compared with animals bearing the MAC16 tumour treated with 0.9% NaCl. [f]$P < 0.005$ compared with animals bearing the MAC16 tumour treated with 0.9% NaCl.

solvent, or of megestrol acetate, on non-tumour-bearing animals (Tables I and IV). Animals bearing the MAC16 tumour were healthy and active, despite the loss of weight, and megestrol acetate had no effect on the general state of health of the animals. Weight gain in animals bearing the MAC16 tumour in response to megestrol acetate was associated with an increase in water intake and an increase in tumour weight. There was no effect of the corn oil alone either on weight loss, or on the growth rate of the tumour (Table IV). Body composition analysis showed a significant reduction in both carcass fat and total non-fat mass in animals bearing the MAC16 tumour (Table V), which was significantly reversed in animals treated with the higher dose of megestrol acetate (300 mg kg$^{-1}$). There was no effect of megestrol acetate on the fat content of non-tumour-bearing animals. The major contributor to the small reduction in host body weight loss in MAC16 tumour-bearing animals treated with the lower dose of megestrol acetate (100 mg kg$^{-1}$) was an increase in the total body water content (Table V). Animals bearing the MAC16 tumour showed a significant reduction in the plasma levels of glucose, FFA and triglycerides compared with non-tumour-bearing controls (Table V). These levels were not altered by injection of the corn oil vehicle alone. However, treatment with megestrol acetate caused a significant increase in blood glucose levels in animals bearing the MAC16 tumour, but had no effect on the plasma levels of either FFA or triglycerides (Table V).

### Discussion

Initial clinical studies have shown megestrol acetate to stimulate appetite and produce weight gain in both cancer patients (Aisner *et al.*, 1988) and in patients with HIV infection (Von Roenn *et al.*, 1988). In both cases, the reported appetite stimulation by megestrol acetate was subjective and no measurements of either caloric input or of body composition were made to determine the body compartment responsible for weight gain. Also a subsequent study of megestrol acetate in a double-blind, placebo-controlled trial, although confirming an increase in appetite, was unable to show an increase in body weight of patients with cancer cachexia (Sleven *et al.*, 1988).

In the present experimental investigation we have confirmed that megestrol acetate is able to reduce the weight loss produced by both TNF and by the MAC16 tumour. Unfortunately, only the acute effects of TNF can be measured, since prolonged administration has been shown to result in the development of tachyphylaxis to the weight losing effect (Mahony & Tisdale, 1988). In both cases this

**Table V** The effect of megestrol acetate on body composition and plasma metabolite levels in animals bearing the MAC16 tumour[a]

| Treatment group[b] | *Body weight (g) (% of body weight)* | *Body fat (g) (% of body weight)* | *Non-fat mass (g) (% of body weight)* | *Glucose* mM | *FFA* mM | *Triglyceride* mM |
|---|---|---|---|---|---|---|
| *A.* | | | | | | |
| 0.9% NaCl | 12.4 ± 0.2 (63.6 ± 0.6) | 1.7 ± 0.1 (8.7 ± 0.7) | 6.4 ± 0.7 (27.7 ± 0.6) | 6.73 ± 0.21 | 0.48 ± 0.07 | 1.72 ± 0.31 |
| 0.9% NaCl + 0.2 ml corn oil | 12.6 ± 0.4 (63.6 ± 0.7) | 1.4 ± 0.1 (6.9 ± 0.5) | 6.1 ± 0.05 (29.5 ± 0.5) | | | |
| 0.9% NaCl + 0.4 ml corn oil | 12.5 ± 0.4 (64.8 ± 0.4) | 0.9 ± 0.06 (6.1 ± 0.4) | 5.6 ± 0.4 (29.1 ± 0.3) | 7.38 ± 0.28 | 0.58 ± 0.03 | 2.08 ± 0.13 |
| 0.9% NaCl + 300 mg kg$^{-1}$ megestrol acetate | 12.6 ± 0.3 (64.8 ± 0.9) | 1.4 ± 0.1 (6.9 ± 0.3) | 5.0 ± 0.3 (28.3 ± 0.9) | 7.01 ± 0.27 | 0.38 ± 0.07 | 1.51 ± 0.04 |
| *B.* | | | | | | |
| 0.9% NaCl | 9.9 ± 0.2 (64.6 ± 0.8) | 0.6 ± 0.06 (3.8 ± 0.3)[e] | 2.9 ± 0.3[e] (31.6 ± 0.8) | 5.07 ± 0.20[e] | 0.25 ± 0.03[c] | 0.30 ± 0.08[d] |
| 0.9% NaCl + 0.2 ml corn oil | 9.4 ± 0.5 (65.1 ± 0.4) | 0.4 ± 0.04 (2.5 ± 0.2) | 4.2 ± 0.9[c] (30.0 ± 0.8) | 3.99 ± 0.66[e] | 0.23 ± 0.03[c] | 0.47 ± 0.03[e] |
| 0.9% NaCl + 100 mg kg$^{-1}$ megestrol acetate | 11.4 ± 0.3 (66.5 ± 1.3)[g] | 0.6 ± 0.07 (3.0 ± 0.5) | 3.0 ± 0.5[c] (30.5 ± 0.5) | 6.25 ± 0.53[f] | 0.33 ± 0.07 | 0.58 ± 0.15[d] |
| 0.9% NaCl + 0.4 ml corn oil | 9.5 ± 0.3 (64.4 ± 0.4) | 0.4 ± 0.1 (3.0 ± 0.5) | 4.2 ± 1.0[c] (32.0 ± 1.0) | 4.71 ± 0.38 | 0.38 ± 0.04 | 0.59 ± 0.09[d] |
| 0.9% NaCl + 300 mg kg$^{-1}$ megestrol acetate | 11.2 ± 0.3 (63.4 ± 0.6) | 1.1 ± 0.06 (6.4 ± 0.3)[h] | 5.2 ± 0.4 (30.0 ± 0.8) | 5.70 ± 0.31[f] | 0.30 ± 0.03 | 0.29 ± 0.12[d] |

[a]Results are mean ± SEM for 10 animals per group. Megestrol acetate was administered s.c. daily over a 7-day period. [b]*A* = non-tumour-bearing. *B* = tumour-bearing. [c]$P < 0.05$ compared with non-tumour-bearing animals treated with 0.9% NaCl. [d]$P < 0.005$ compared with non-tumour-bearing animals treated with 0.9% NaCl. [e]$P < 0.001$ compared with non-tumour-bearing animals treated with 0.9% NaCl. [f]$P < 0.05$ compared with tumour-bearing animals treated with 0.9% NaCl. [g]$P < 0.005$ compared with tumour-bearing animals treated with 0.9% NaCl. [h]$P < 0.001$ compared with tumour-bearing animals treated with 0.9% NaCl.

was associated with a significant increase in both food and water intake, thus confirming the appetite stimulatory effect of megestrol acetate. Megestrol acetate was unable to prevent the loss in body weight in animals given the same amount of food and water as the TNF-treated animals and thus weight gain must arise solely from the increased intake.

We have shown previously that the weight loss induced in mice by TNF is, at least in part, due to dehydration (Mahony & Tisdale, 1989), and this is confirmed in the present experiments. The TNF-induced weight loss is also accompanied by a decrease in carcass fat with no effect on the non-fat body mass. Weight reversal in TNF-treated animals by megestrol acetate is accompanied by an increase in carcass water content, with no effect on carcass fat, and the weight reversal appears to be solely due to rehydration. The reduction in blood glucose produced by TNF is also significantly reversed by megestrol acetate but the decrease in plasma FFA and increase in plasma triglycerides are not affected. Hyperlipidaemia produced by TNF is thought to arise as a result of the inhibition of adipose tissue lipoprotein lipase activity and has been considered important in the cachexia ( Beutler & Cerami, 1987). However, recent evidence suggests that inhibition of adipose tissue lipoprotein lipase is not required for TNF-induced hyperlipidaemia (Feingold *et al.*, 1989) and that TNF-induced hyperlipidaemia is not inevitably linked to the syndrome of cachexia (Grunfeld *et al.*, 1989) and this would be supported by the results of the present experiments.

Weight gain in animals bearing the MAC16 also arises from an increase in body water content at the lower dose of megestrol acetate, although both the carcass fat and non-fat mass is also significantly increased at the higher dose of megestrol acetate. Blood glucose levels are also increased by megesterol acetate as for TNF-treated animals. However, the increase in host body weight is also accompanied by a highly significant increase in tumour dry weight, possibly due to the increased blood glucose level. As with TNF-treated animals, there is no effect on the plasma levels of FFA or triglycerides. Tumours from animals administered megestrol acetate had the same water content (83 ± 1.8%) as did controls (81 ± 0.5%) and the increase in tumour weight arose from an increase in cellularity of the tumours.

In many of its effects, megestrol acetate behaves like an adrenal cortical steroid with increased water retention, due to expansion of the extracellular volume, and an increased blood glucose level, which may arise from a decreased peripheral utilisation of glucose, together with an increased glucose release from the liver. The effect of megestrol acetate on host body weight loss in animals bearing the MAC16 tumour is similar to that of insulin, where weight reversal is also seen with an accompanying increase in tumour weight (Beck & Tisdale, 1989), and differs from that of fish oil, where complete prevention of the loss of host body weight occurs with a concomitant reduction in tumour weight (Tisdale & Dhesi, unpublished results). Thus, when choosing an appropriate anticachectic treatment both the analysis of the body compartment responsible for weight gain and the effect of the agent on the growth of the tumour are important parameters, in addition to the weight gain of the patients.

The authors would like to thank Mr M. Wynter for the tumour transplantation. This work has been supported by a grant from the Cancer Research Campaign.

### References

AISNER, J., TCHEKMEDYIAN, S., TAIT, N., PARNES, H. & NOVAK, M. (1988). Studies of high-dose megestrol acetate: potential applications in cachexia. *Seminars in Oncology*, **15** (suppl. 1), 68.

BECK, S.A. & TISDALE, M.J. (1987). Production of lipolytic and proteolytic factors by a murine tumor-producing cachexia in the host. *Cancer Res.*, **47**, 5919.

BECK, S.A. & TISDALE, M.J. (1989). Effect of insulin on weight loss and tumour growth in a cachexia model. *Br. J. Cancer*, **59**, 677.

BEUTLER, B. & CERAMI, A. (1987). Cachectin: more than a tumor necrosis factor. *N. Engl. J. Med.*, **316**, 379.

BIBBY, M.C., DOUBLE, J.A., ALI, S.A., FEARON, K.C.H., BRENNAN, R.A. & TISDALE, M.J. (1987). Characterisation of a transplantable adenocarcinoma of the mouse colon producing cachexia in recipient animals. *J. Natl Cancer Inst.*, **78**, 539.

CHLEBOWSKI, R.T. (1985). Significance of altered nutritional status in acquired immune deficiency syndrome (AIDS). *Nutr. Cancer*, **7**, 85.

DE WYS, W. (1985). Management of cancer cachexia. *Seminars in Oncology*, **12**, 452.

FEINGOLD, K.R., SOUED, M., STAPRANS, I. & 6 others (1989). Effect of tumor necrosis factor (TNF) on lipid metabolism in the diabetic rat. Evidence that inhibition of adipose tissue lipoprotein lipase activity is not required for TNF-induced hyperlipidaemia. *J. Clin. Invest.*, **83**, 1116.

GRUNFELD, C., WIKING, H., NEESE, R. & 5 others (1989). Persistence of the hypertriglyceridemic effect of tumor necrosis factor despite development of tachyphylaxis to its anorectic/cachectic effect in rats. *Cancer Res.*, **49**, 2554.

HEBER, D., BYERLEY, L.O., CHI, J. & 4 others (1986). Pathophysiology of malnutrition in the adult cancer patient. *Cancer*, **58**, 1867.

LAHDEVIRTA, J., MAURY, C.P.J., TEPPO, A.-M. & REPO, H. (1988). Elevated levels of circulating cachectin/tumor necrosis factor in patients with acquired immunodeficiency syndrome. *Am. J. Med.*, **85**, 289.

LUNDHOLM, K., EDSTRÖM, S., KARLBERG, J., EKMAN, L. & SCHERSTEN, T. (1980). Relationship of food intake, body composition and tumor growth to host metabolism in non-growing mice with sarcoma. *Cancer Res.*, **40**, 2515.

MAHONY, S.M. & TISDALE, M.J. (1988). Induction of weight loss and metabolic alterations by human recombinant tumour necrosis factor. *Br. J. Cancer*, **58**, 345.

MAHONY, S.M. & TISDALE, M.J. (1989). Reversal of weight loss induced by tumor necrosis factor-alpha. *Cancer Lett.*, **45**, 167.

MAHONY, S.M., BECK, S.A. & TISDALE, M.J. (1988). Comparison of weight loss induced by recombinant tumour necrosis factor with that produced by a cachexia-inducing tumour. *Br. J. Cancer*, **57**, 385.

OLIFF, A. (1988). The role of tumor necrosis factor (cachectin) in cachexia. *Cell*, **54**, 141.

SOCHER, S.H., MARTINEZ, D., CRAIG, J.B., KUHN, J.G. & OLIFF, A. (1988). Tumor necrosis factor not detectable in patients with clinical cancer cachexia. *J. Natl Cancer Inst.*, **80**, 595.

SLEVEN, M.L., JOEL, S.P., STUBBS, L. & 4 others (1988). A randomised double blind placebo controlled trial of medroxyprogesterone acetate (MPA) in cancer cachexia. *Proc. Soc. Clin. Oncol.*, **7**, 283.

VON ROENN, J.H., MURPHY, R.L., WEBER, K.M., WILLIAMS. L.M. & WEITZMAN, S.A. (1988). Megesterol acetate for the treatment of cachexia associated with human immunodeficiency virus (HIV) infection. *Ann. Int. Med.*, **109**, 840.